# Who leaves a suicide note? A systematic review of differences between note writers and non‐note writers

**DOI:** 10.1002/pcn5.70414

**Published:** 2026-09-30

**Authors:** Giulia Gotti, Federica Turolla, Alessandra Bordessa, Claudia Bruni, Fabio Madeddu, Lorenzo Montali, Guendalina Gentile, Riccardo Zoja, Riccardo Primavera, Stefano Tambuzzi, Cristina Cattaneo, Raffaella Calati

**Affiliations:** ^1^ Department of Psychology, University of Milano‐Bicocca Milan Italy; ^2^ IGF, University of Montpellier, CNRS, INSERM Montpellier France; ^3^ Family Counseling Services, Azienda Socio‐Sanitaria Territoriale (ASST) of Pavia Pavia Italy; ^4^ Department of Biomedical Sciences for Health Section of Legal Medicine, University of Milan Milan Italy; ^5^ LABANOF, Laboratory of Forensic Anthropology and Odontology, Institute of Legal Medicine, Department of Biomedical Sciences for Health University of Milan Milan Italy

**Keywords:** non‐note writers, note writers, suicide note

## Abstract

Suicide notes represent one of the few first‐person sources of information on the suicidal process. However, only a minority of individuals who die by suicide leave a note, raising the question of whether note writers (NW) are representative of the broader population of suicide decedents. This systematic review aimed to synthesize the available evidence on differences between NW and non‐note writers (NNW) across sociodemographic, clinical, psychosocial, and suicide‐related variables. A systematic search was conducted in PubMed and ProQuest databases up to June 22, 2026, following PRISMA guidelines. Studies were included if they compared NW and NNW on at least one variable, or investigated predictors of note‐leaving. Thirty‐three studies met the inclusion criteria. Evidence was heterogeneous and suggested that NW cannot be clearly distinguished from NNW based on sociodemographic and clinical variables. Concerning suicide‐related variables, the method of suicide was the most consistently different between NW and NNW; in particular, the most frequent methods of suicide in NW, compared to NNW, were non‐violent methods like poisoning/overdose and gas inhalation. Rather than solely reflecting a distinct psychological profile, suicide note‐leaving may also be influenced by the circumstances surrounding the suicidal act. One possible, although untested, explanation is that leaving a note requires a degree of cognitive and emotional organization that may be less compatible with highly disorganized or impulsive suicidal states.

## Graphical abstract

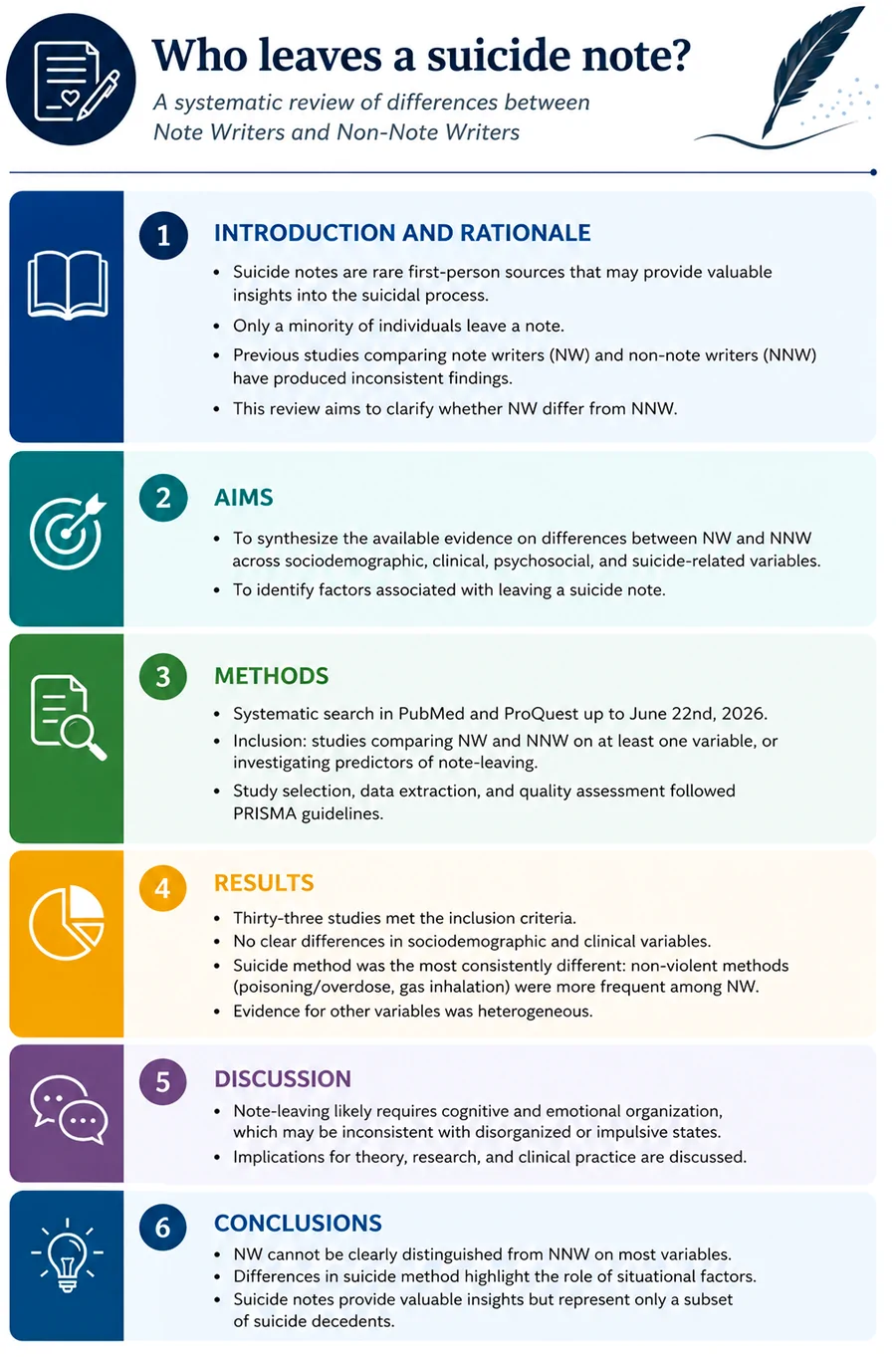

## INTRODUCTION

Suicide is a major public health concern worldwide, accounting for more than 720,000 deaths each year.^1^ Despite extensive research efforts, understanding the factors associated with suicide remains challenging, particularly because the subjective experiences that precede suicide death cannot be directly investigated.^2^ To address this limitation, researchers have relied on a range of postmortem sources, including psychological autopsies, medical and psychiatric records, and interviews with relatives and acquaintances.

Among these sources, suicide notes occupy a unique position. Unlike other postmortem data, which are reconstructed retrospectively by third parties, suicide notes represent first‐person communications written by individuals shortly before their death. As such, they may provide valuable insights into the thoughts, emotions, motivations, and interpersonal concerns that accompanied the suicidal process. Previous research suggests that suicide notes may reveal intentions, psychological pain, hopelessness, perceived burdensomeness, interpersonal difficulties, and meaning‐making processes that are often inaccessible through other postmortem methods.^3^, ^4^ Moreover, findings from suicide note analyses have informed several influential theories of suicide, including conceptualizations emphasizing psychache and interpersonal processes.^5^, ^6^ Although suicide notes are often examined within the broader framework of psychological autopsy investigations,^7^ they constitute one of the few sources that directly reflect the deceased individual's own perspective.

A key methodological question therefore concerns whether individuals who leave a suicide note are representative of all suicide decedents. If note writers (NW) differ systematically from non‐note writers (NNW), conclusions drawn from suicide note analyses may reflect the characteristics of a selected subgroup rather than those of suicide decedents as a whole. Available evidence indicates substantial variability across studies in the prevalence of suicide NW. In large‐scale studies, prevalence estimates ranged from 18.25%^8^ to 31%^9^ in the United States (with sample sizes ranging from 2936 to 32,151 suicides), while a prevalence of 30.1% was reported in Japan (based on a sample of 5161 suicides^10^). Because only a minority of individuals who die by suicide leave a note, findings derived from suicide note analyses may not necessarily generalize to the broader population of suicide decedents. Previous studies comparing NW and NNW have yielded inconsistent findings regarding sociodemographic characteristics, psychiatric history, stressful life events, and suicide‐related factors. Clarifying these differences is essential both for interpreting the existing suicide note literature and for determining the extent to which suicide notes can be used to understand suicide risk more generally.

This systematic review aimed to identify and synthesize the evidence regarding differences between NW and NNW. Specifically, we examined (a) sociodemographic characteristics (e.g., sex, age, and marital status), (b) clinical and psychosocial factors (e.g., mental and physical illnesses, alcohol, and substance use), and (c) suicide‐related variables (e.g., suicide method and previous suicide attempt(s) (SA)) associated with the presence of a suicide note.

## METHODS

This systematic review was performed according to the PRISMA (Preferred Reporting Items for Systematic Reviews and Meta‐Analysis) statement.^11^ The use of ChatGPT in the review process was documented in accordance with the principles of the PRISMA‐trAIce reporting framework.^12^

A literature search was conducted using PubMed and ProQuest databases (up to June 22, 2026), searching across all ProQuest subject collections available through the institutional subscription (MEDLINE, PTSDpubs, Periodicals Index Online, International Bibliography of the Social Sciences (IBSS), ProQuest Dissertations & Theses Global, Linguistics and Language Behavior Abstracts (LLBA), Library & Information Science Abstracts (LISA), Natural Science Collection, Education Research Index, and Social Science Premium Collection), and Rayyan software was employed to identify and exclude duplicates across the two databases. The search strings are reported in Supporting Information S1: Appendix 1. Articles were independently screened for eligibility by three authors (GG, AB, and RC). In cases of disagreement, the three reviewers discussed the issue until an agreement was reached.

Studies were eligible if they compared NW and NNW with respect to sociodemographic, clinical, psychosocial, or suicide‐related characteristics, or examined factors associated with the likelihood of leaving a suicide note. Studies were included if written in English, Italian, French, German, and Spanish.

Studies were excluded if they did not include NW and NNW, compared real with simulated suicide notes, or compared different types of notes (e.g., notes from suicides vs. notes from suicide attempters). Reviews, meta‐analyses, editorials, protocols, and dissertations were excluded. In case of studies performed on overlapping samples, the study with the wider sample was included (e.g., Salib et al.^13^ and Salib and Maximous^14^ were performed on overlapping samples, thus only^14^ was included).

From each included article, GG and AB independently extracted the main characteristics: authors and year of publication, setting, type of population, sample size, and percentage of NW and NNW, type of statistical analysis, investigated variables, and main results. See Tables 1a and 1b for study characteristics.

**Table 1a:** Characteristics of studies whose outcome was suicide.

Authors and year	Setting	Type of population	Sample size (*N* and % of NW)	Investigated variables*	Main results
Beck et al.^15^	Philadelphia, Pennsylvania, U.S.A., 1972	People who died by or attempted suicide	*N* = 194 (24%)	Depression, hopelessness, suicide intent (31 variables).	NW had more previous SA than NNW and had higher suicidal intent.
Callanan & Davis^16^	Ohio, U.S.A., 1997–2006	People who died by suicide	*N* = 621 (231, 37.2%)	Sex, age, marital status, employment status, living arrangements, ethnicity.	NW were more likely to have lived alone and were less likely to have made prior suicide threats.
Carpenter et al.^17^	Queensland, Australia	People who died by suicide	*N* = 533 (325, 61%)	Sex, age, socioeconomic status, ethnicity, mental health issues, method.	NW were less likely to be females, Aboriginal Australians, and to use a vehicle or train as a method of suicide, and more likely to use gas as a method of suicide.
Cerel et al.^8^	Kentucky, U.S.A.	People who died by suicide	*N* = 2936 (536, 18.25%)	Sex, age, marital status, educational level, homeless status, ethnicity, pregnancy status, veteran status, mental health problems/treatment, alcohol/substance abuse, physical health problem, method used in the suicide, place of injury, whether the decedent was injured at home, disclosed suicide intent, history of SA, life crisis, relationship problems, family stressor, legal problem, job problem, financial problem, school problem, suicide of friend/family, death of friend/family perpetrator/victim of interpersonal violence recent.	No differences in demographics of circumstances of death were found between NW and NNW.
Chavez‐Hernandez et al.^18^	Mexico, 1995–2001	People who died by suicide	*N* = 733 (106, 14.46%) (*N* = 106 NNW were included in the analysis)	Sex, age, educational status, occupation, zone of residence, religious belief, marital status, place of suicide, suicide method, previous SA.	NW had more frequently completed high school and/or college, lived in urban areas than NW, and had less previous SA than NNW.
Cheung et al.^19^	New Zealand	Old people >65 who died by suicide	*N* = 225 (88, 41.5%)	Gender, age, marital status, living situation, ethnicity, psychiatric conditions, medical conditions, past SA, recent contact with psychiatric services, alcohol level at postmortem, method, location.	Females and Caucasians were more likely to be NW and to use non‐violent methods.
Chia et al.^20^	Singapore, 2000–2004	People who died by suicide	*N* = 172 (398, 23%)	Gender, age, marital status, occupation, ethnicity, place of birth (local vs overseas), mental illness, physical illness, prior admission to mental/general hospital, socioeconomic problems (relationship, financial, employment, legal, drug, gambling), method.	NW were more likely to be younger, local‐born and single, to have gambling, financial, employment or relationship problems, and less likely to have drug‐related, mental or physical illnesses; tended to choose suicide methods that required more planning (e.g., hanging, poison or gas) and tended to choose less jumping from high buildings.
Chynoweth^21^	Canberra, Australia, 1972–1973	People who died by suicide	*N* = 135 (27, 20%)	Sex, age, marital status, and method of suicide.	NW were more likely to have used firearms.
Cohen & Fielder^22^	Pittsburgh, Pennsylvania, U.S.A.	People who died by suicide or attempted suicide	*N* = 1033 (220, 21.30%)	Sex, age, marital status, ethnicity, method of suicide, multiplicity of suicidal incidents.	NW were more likely to be females, white, and non‐widowed; as for method, they were more likely to use drugs; females were more likely to choose asphyxiation; firearms were more likely for widowed people and less likely for married people.
Demirel et al.^23^	Ankara, Turkey, 2005	People who died by suicide	*N* = 148 (49, 34.5%)	Marital status, children, living conditions (living with family/alone), occupation, psychiatric disorder, physical disorder, precipitating factor, previous SA.	NW had a lower rate of psychiatric disorders; poor health was the most common reason for NNW, while financial‐occupational problems were the most common ones for NW.
Eisenwort et al.^24^	Vienna, Austria, 2002–2003	People who died by suicide	*N* = 173 (53, 30.64%)	Sex, age, marital status, occupation, psychiatric care, motive, method.	NW did not differ from NNW.
Foster^25^	Northern Ireland	People who died by suicide	*N* = 118 (38%)	Sex, age, marital status, living alone, occupation, contact with healthcare professionals during the last year, current psychotropic medication, current physical illness, current disorders, previous history of self‐harm, occurrence of life events during the last year of life, methods of suicide, unemployment.	NW did not differ from NNW.
Freuchen & Groholt^26^	Norway, 2007–2009	Children and young adolescents (<15)	*N* = 42 (18, 43%)	Sex, age, method, time of day, bright/dark season, mental health problems, contact with mental health services, personality traits, suicidal behavior, loss, conflict, bullied, altered behavior.	NW more frequently had previous SA, and had shown altered behavior prior to suicide (being helpful, cuddly, silent, joyful, tense or relaxed) than NNW.
Girdhar et al.^27^	New Delhi, India, 2001–2002	People who died by suicide	*N* = 320 (50, 15.6%) (50 NNW were included in the analysis)	Sex, age, educational status, socioeconomic status, occupation, marital status, religious belief, physical illness, psychiatric illness treated, previous SA, method.	NW were more frequently males and had higher education (graduate or above); they were more frequently students or workers, not married, and religious.
Haines et al.^28^	Tasmania, Australia, 20‐year period	People who died by suicide	*N* = 1051 (347, 33%)	Sex, age, marital status, living arrangements, employment, medical and psychiatric history, suicide history and stated intention, psychological symptoms in the days and weeks leading up to the act, psychological state prior to the act, reasons for the suicide, method of suicide.	NW were more likely to be divorced, live alone, be younger, distressed, hypochondriacs, and have interpersonal loss/conflict and extrapunitive reasons as precipitating factors. More frequent methods in NW were gas inhalation, firearms, and medication. NW were less likely to have contacted their family doctor and to be under medical/psychiatric supervision, less past hospitalization, less confused at the time of the act, less psychotic symptoms, less psychiatric disturbance as reason, method: less poison, hanging.
Ho et al.^29^	Hong Kong, 1992	People who died by suicide	*N* = 769 (154, 20%)	Sex, age, marital status, occupation, religious beliefs, living alone, physical illness, psychiatric illness, previous SA, weekday of suicide, suicide method.	NW were more likely to be young females, non‐widowed, have no previous SA, have no previous psychiatric illness, and have religious beliefs. Those whose principal job was doing household duties tended to be NNW. Subjects who used easily available methods which required no preparation (e.g., jumping from a height, cutting wrists) tended to leave fewer suicide notes.
Karbeyaz et al.^30^	Eskişehir, Turkey, 2001–2011	People who died by suicide	*N* = 399 (168, 42.1%)	Gender, age, occupation.	NW were younger (<35 years old).
Kuwabara et al.^10^	Kobe City, Japan, 1981–2001	People who died by suicide	*N* = 5161 (1553, 30%)	Gender, age, marital status, residential status, suicide method, reason for suicide, day and time of death, location, previous visits to psychiatric clinic, previous suicidal behavior, physical disease, results of toxicological examination.	NW were more likely to be female, to live alone, and to use carbon monoxide, hanging, or sharp instruments as methods of suicide; they were less likely to have physical illnesses and/or psychiatric disorders as reasons or unknown reasons for suicide, and/or history of psychiatric disorders.
Lang et al.^31^	Southern Germany	People who died by suicide	*N* = 612 (255, 42%)	Gender, age, marital status, psychiatric issues, alcohol and substance abuse, stressful life events, methods of suicide.	NW were more often female, less often married, more often in a financial or partnership crisis, more likely to have a medical illness, more often used poison, and less likely to have a history of alcohol abuse disorder.
Li et al.^32^	Pudong, Shanghai, China, 2004–2016	People who died by suicide	*N* = 2407 (458, 19%)	Age, education level, place of origin (local vs migrants), physical illness, psychiatric illness, history of self‐harm, in debt, methods of suicide.	NW were more likely to be males, older males, younger women, use gas poisoning, followed by liquid/solid poisoning and hanging.
Mejías‐Martín et al.^33^	Granada, Spain, 2007–2013	People who died by suicide	*N* = 286 (105, 36.7%)	Age, sex, method of suicide, year and month of suicide.	NW more frequently used poisoning as method.
Men et al.^34^	Hong Kong, 2012–2017	Cancer patients who died by suicide	*N* = 551 (181, 32.85%)	Age, sex, marital status, employment status, type of housing, living alone, psychiatric illness, other physical illness besides cancer, methods of suicide, time and place of suicide.	NW were younger, more likely to be male, use non‐violent suicide methods, have better housing conditions, and live alone.
O'Connor et al.^35^	Northern Ireland, 1993–1994	People who died by suicide	*N* = 142 (54, 38%)	Sex, age, marital status, social class (type of occupation), method.	NW were more frequently men, used more poisoning and hanging, and less drowning as a method.
O'Rourke et al.^36^	37 U.S. states, 2013–2017	People who died by suicide	*N* = 98 515 (30 477, 30.94%)	Gender identity, age, marital status, education, ethnicity, military status, alcohol or substance use, mental health variables (SI and SA history)	NW were more likely to be part of a sexual/gender minority, female, older, non‐Hispanic white, to have higher education level, history of SI and SA; not having alcohol problems predicted leaving suicide notes.
Posener et al.^37^	Montreal, Canada 1978–1982	Adolescents (10–19 years old) who died by suicide	*N* = 136 (13, 10%)	Sex, age, method.	Shooting was more common among NW.
Rockett et al.^9^	U.S. states, 2011–2013	People who died by suicide	*N* = 32 151 (31%)	Sex, age, marital status, education, ethnicity, military veteran, mental diagnosis, mental health treatment, physical health problem, personal problems, recent crisis, alcohol or substance use (blood alcohol concentration, drug positives), suicide method, region of death, county urbanicity, county poverty.	NW were more likely to use drug intoxication and other poisoning suicides than firearms.
Salib & Maximous^14^	Cheshire, 1989–2001	Elderly who died by suicide	*N* = 216 (71, 33%)	Sex, marital status, children, living alone, known to services, suicide place, method.	NW were less likely to be known to psychiatric services, more likely to use overdose, plastic bags, electrocution, or car exhaust, and less likely to use drowning as a method.
Stack & Rockett^38^	17 U.S. states, 2003–2006	People who died by suicide	*N* = 30 570 (9048, 29.6%)	Gender, age, marital status, ethnicity, urban residence, psychiatric condition, substance/alcohol abuse, stressful life events (death of a significant other, physical health issue, financial problems, job problems, intimate partner problem eg. domestic violence, other relationship problems, legal problems, recent crisis), method of suicide.	NW were more likely to be females, Caucasian, not married, middle‐aged, have financial problems, relationship problems, illness, job problems, grieving a significant other, have psychiatric issues, alcohol abuse, use poison, drowning, firearms, and hanging as method.
Tewksbury et al.^39^	(Louisville, Kentucky, 2003–2008	Males who died by suicide	*N* = 419 (89, 26.3%)	Sex, ethnicity, age, marital status, employment status, academic achievement, BMI, psychological problems, prior SA or SI, declared suicide intentions, evidence of planning, alcohol use prior to suicide, place of suicide, method.	NW were more likely to be older men and less likely to use guns.
Weeke & Oberwittler^40^	Germany	Homicide‐Suicide cases (or attempted)	*N* = 288 (119, 41.32%)	Sex, age, marital status, academic achievement, psychological problems, prior SA or SI, declared suicide intentions, evidence of planning.	NW were more likely to be older, have declared suicide intentions, show evidence of planning, have underage children, and have killed a child, especially when underage or living in the same household, in a homicide‐suicide episode.
Wong et al.^41^	34 U.S. states, 2003–2017	Asian and Pacific Islander Americans who died by suicide	*N* = 3 657 (1130, 31%)	Gender, age, nativity (immigrant vs U.S.‐born).	Females and youths (<25 years old) had higher odds of leaving suicide notes.

Abbreviations: BMI, Body Mass Index; NNW, Non‐Note Writers; NW, Note Writers; SA, Suicide Attempt; SI, Suicidal Ideation.

* Variable labels are reported as originally used in the included studies.

**Table 1b:** Characteristics of studies whose outcome was suicide attempt.

Authors and year	Setting	Type of population	Sample size (*N* and % of NW)	Investigated variables*	Main results
Beck et al.^15^	Philadelphia, Pennsylvania, U.S.A., 1972	People who died by or attempted suicide	*N* = 231 (14%)	Depression, hopelessness, suicide intent (57 variables).	NW less often abused drugs, had higher vocabulary scores than NNW, and had higher suicidal intent.
Huppert et al.^42^	U.S.A	Soldiers and marines with SI and/or behavior	*N* = 657 (112, 17%)	Gender, age, marital status, parental status, military career variables, SA during SI.	NW had an increased likelihood of making a SA and higher lethality of SA during episodes of SI.
Ruiz et al.^43^	Buenos Aires, Argentina, 1999–2003	Older suicide attempt inpatients (>65 years old)	*N* = 31 (12, 38.71%)	Sex, age, marital status, religion, living alone, level of education, present psychiatric treatment, depression, hopelessness, SI, previous suicidal arousal, afterwards suicidal arousal, severity of SA, dementia, narcissistic personality disorder, physical pain, previous SA, family history of suicide or SA.	NW showed higher severity of SA and higher hopelessness level.
Weeke and Oberwittler^40^	Germany	Homicide‐Suicide cases (or attempted)	*N* = 288 (119, 41.32%)	Sex, age, marital status, academic achievement, psychological problems, prior SA or SI, declared suicide intentions, evidence of planning.	NW were more likely to be older, have declared suicide intentions, have shown evidence of planning, have underage children, have killed a child, especially when underage or living in the same household, in a homicide‐suicide episode.

Abbreviations: NNW, Non‐Note Writers; NW, Note Writers; SA, Suicide Attempt; SI, Suicidal Ideation.

* Variable labels are reported as originally used in the included studies.

### Quality of reporting assessment

The quality of reporting was assessed using the Quality Assessment Tool for Observational Cohort and Cross‐Sectional Studies developed by the National Heart, Lung, and Blood Institute (NHLBI). Items that were not applicable to the study design (e.g., exposure assessed prior to outcome, repeated exposure assessment, or loss to follow‐up) were coded as “Not Applicable” (NA) and excluded from the overall score calculation. Overall study quality was classified as good, fair, or poor based on the proportion of positive ratings among applicable items (excluding NA responses): Good = ≥75% “Yes” responses; Fair = 50%–74% “Yes” responses; Poor = <50% “Yes” responses.

An independent quality assessment was first performed by GG using the NHLBI Quality Assessment Tool. Subsequently, an AI‐assisted assessment was conducted using ChatGPT (OpenAI GPT‐5.5). Full‐text articles, together with the NHLBI checklist and the explanation of each item, were provided to ChatGPT, which generated a preliminary quality assessment for each study. The independent assessment performed by GG and the AI‐generated assessments were then compared. Any discrepancies were reviewed and resolved through discussion among the authors, resulting in the final quality ratings. ChatGPT was used solely as a decision‐support tool, and the final quality ratings were determined by the authors. At every stage, the AI‐generated ratings were treated as provisional suggestions rather than final judgments: each rating was reviewed by GG against the source article, any disagreement with the AI output was discussed among the authors, and the human‐agreed rating, not the AI output, was recorded as the final score. No quality rating was accepted without independent human verification.

Because most studies were cross‐sectional, retrospective, registry‐based, or based on suicide note content analyses, several items of the NHLBI tool were frequently not applicable. General decision rules: Q1 (Research question): scored “Yes” when the study objective or research question was explicitly stated; Q2 (Study population): scored “Yes” when the population and source of cases were clearly described; Q3 (Participation rate ≥50%): scored “Yes” only when the study included a clearly defined population‐based sample or when participation/completeness could reasonably be assumed. If information was unavailable, “Cannot Determine” (CD) was assigned; Q4 (Selection criteria): scored “Yes” when inclusion/exclusion criteria or case definitions were described; Q5 (Sample size justification/power): scored “Yes” only when an explicit power analysis, sample size justification, or variance/effect estimate was reported. Large sample size alone was not sufficient; Q6 (Exposure measured before outcome): generally coded “NA” because most studies were cross‐sectional or retrospective; Q7 (Sufficient timeframe): generally coded “NA” for cross‐sectional studies without temporal follow‐up; Q8 (Different levels of exposure): scored “Yes” when exposures were analyzed across categories, groups, or gradients; Q9 (Exposure measures valid and reliable): scored “Yes” when exposure variables were clearly defined and obtained through standardized records, validated instruments, official databases, or systematic coding procedures; Q10 (Exposure assessed more than once): generally coded “NA” because repeated assessments were uncommon; Q11 (Outcome measures valid and reliable): scored “Yes” when outcomes were clearly operationalized and consistently measured across participants; Q12 (Blinding): usually coded “CD” because blinding procedures were rarely reported; Q13 (Loss to follow‐up): generally coded “NA” because follow‐up was not part of most study designs; Q14 (Confounding): scored “Yes” only when multivariable analyses or statistical adjustment for confounders were performed. Descriptive analyses without adjustment were coded “No.”

The preliminary agreement between GG and the AI‐assisted assessment was high, with concordant ratings for 435 of 462 checklist items (94.2%). Only 27 ratings (5.8%) required discussion. Nearly half of all discrepancies (48.1%) concerned Item 5 (sample size justification/power calculation), whereas the remaining disagreements were mainly related to participation rate (Item 3), selection criteria (Item 4), validity of exposure measures (Item 9), and adjustment for confounding (Item 14). All discrepancies were subsequently resolved by consensus among the authors.

## RESULTS

The search initially identified 870 references; after eliminating duplicates with Rayyan software, 439 papers were screened for eligibility. Thirty‐three studies met the inclusion criteria and were included. See Supporting Information S2 for the study flowchart.

For the purpose of synthesis, variables were grouped into three broad domains: (a) sociodemographic variables, (b) clinical and psychosocial variables, and (c) suicide‐related variables.

Sociodemographic variables included sex, age, marital status, children, education, occupation, socioeconomic status, living condition (living alone or with someone else, and living in an urban or rural zone), ethnicity, country of origin (locally born or immigrant), sexual orientation and gender identity, religious beliefs, and military career variables (veteran status and military service characteristics).

Clinical and psychosocial variables included mental and physical health conditions, contact with healthcare services, substance use, stressful life events (e.g., relationship, financial, occupational, legal, or school‐related problems), interpersonal violence, recent crises, and other psychological characteristics.

Suicide‐related variables included method, past suicidal ideation (SI), past SA, place, time, having disclosed suicide intent to others, evidence of planning, family history of suicide or SA, precipitating circumstances, psychological state and behavior before the suicide or SA, severity of SA, and alcohol or drug use at the time of suicide.

Some studies had suicide as the main outcome, some had SA, and some had both. Results are reported separately for suicides and SA.

### Suicide

Twenty‐nine of the 33 included studies (87.9%) investigated suicide deaths. One study (Weeke & Oberwittler^40^) included both suicide deaths and SA but did not report the findings separately according to the outcome. Consequently, whenever this study is cited in the present review, the reported findings should be interpreted as referring to a mixed sample of suicide deaths and SA.

### Sociodemographic variables

The most frequently investigated sociodemographic variables were sex (*k* = 28, 84.8%), age (*k* = 28, 84.8%), marital status (*k* = 21, 63.6%), housing conditions (*k* = 13, 39.4%), occupation (*k* = 11, 33.3%), ethnicity (*k* = 9, 27.3%), and education level (*k* = 6, 18.2%). Sociodemographic variables examined in fewer than 5 studies are summarized in Table 2a.

**Table 2a:** Sociodemographic, clinical, psychosocial, and suicide‐related variables investigated in note writers (NW) and non‐note writers (NNW) in studies included in the systematic review with suicide death as outcome.

Variables	Studies that investigated the variable (*k*, %)	Significant differences between NW and NNW
**Sociodemographic variables**		
Gender	*k* = 28, 84.8%	**NW were more likely to be women in *k* = 8**: Cheung et al.^19^ Cohen & Fiedler^22^ Ho et al.^29^ (only among youth) Kuwabara et al.^10^ Lang et al.^31^ O'Rourke et al.^36^ Stack & Rockett^38^ Wong et al.^41^ **NW were more likely to be men in *k* = 6**: Carpenter et al.^17^ Girdhar et al.^27^ Li et al.^32^ Men et al.^34^ O'Connor et al.^35^ Tewksbury et al.^39^ (only among older)
	Callanan & Davis^16^	
	Carpenter et al.^17^	
	Cerel et al.^8^	
	Chavez‐Hernandez et al.^18^	
	Cheung et al.^19^	
	Chia et al.^20^	
	Chynoweth^21^	
	Cohen & Fiedler^22^	
	Eisenwort et al.^24^	
	Foster^25^	
	Freuchen & Grøholt^26^	
	Girdhar et al.^27^	
	Ho et al.^29^	
	Haines et al.^28^	
	Karbeyaz et al.^30^	
	Kuwabara et al.^10^	
	Lang et al.^31^	
	Mejías‐Martín et al.^33^	
	Men et al.^34^	
	O'Connor et al.^35^	
	O'Rourke et al.^36^	
	Posener et al.^37^	
	Rockett et al.^9^	
	Salib & Maximous^14^	
	Stack & Rockett^38^	
	Tewksbury et al.^39^	
	Weeke & Oberwittler^40^	
	Wong et al.^41^	
Age	*k* = 28, 84.8%	**NW were more likely to be younger in *k* = 7**: Chia et al.^20^ Ho et al.^29^ (only among women) Haines et al.^28^ Karbeyaz et al.^30^ Li et al.^32^ (only among women) Men et al.^34^ Wong et al.^41^ (<25) **NW were more likely to be middle‐aged in *k* = 1:** Stack & Rockett^38^ **NW were more likely to be older in *k* = 4**: Li et al.^32^ (only among men) O'Rourke et al.^36^ Tewksbury et al.^39^ (only among men) Weeke & Oberwittler^40^
	Callanan & Davis^16^	
	Carpenter et al.^17^	
	Cerel et al.^8^	
	Chavez‐Hernandez et al.^18^	
	Cheung et al.^19^	
	Chia et al.^20^	
	Chynoweth^21^	
	Cohen & Fiedler^22^	
	Eisenwort et al.^24^	
	Foster^25^	
	Freuchen and Grøholt^26^	
	Girdhar et al.^27^	
	Ho et al.^29^	
	Haines et al.^28^	
	Karbeyaz et al.^30^	
	Kuwabara et al.^10^	
	Lang et al.^31^	
	Li et al.^32^	
	Men et al.^34^	
	Mejías‐Martín et al.^33^	
	O'Connor et al.^35^	
	O'Rourke et al.^36^	
	Posener et al.^37^	
	Rockett et al.^9^	
	Stack and Rockett^38^	
	Tewksbury et al.^39^	
	Weeke & Oberwittler^40^	
	Wong et al.^41^	
Marital status	*k* = 21, 63.6%	**NW were more likely to be non‐married in *k* = 5:** Chia et al.^20^ (single) Haines et al.^28^ (divorced) Lang et al.^31^ (less married) Stack and Rockett^38^ (not married) Girdhar et al.^27^ (divorced/separated/widowed) **NW were more likely to be non‐widowed in *k* = 2**: Cohen & Fiedler^22^ (single, married, separated females vs widowed) Ho et al.^29^ (non‐widowed)
	Callanan & Davis^16^	
	Cerel et al.^8^ Chavez‐Hernandez et al.^18^	
	Cheung et al.^19^	
	Chia et al.^20^	
	Chynoweth^21^	
	Cohen & Fiedler^22^	
	Demirel et al.^23^	
	Eisenwort et al.^24^	
	Foster^25^	
	Ho et al.^29^	
	Haines et al.^28^	
	Kuwabara et al.^10^	
	Lang et al.^31^	
	Men et al.^34^	
	O'Connor et al.^35^	
	Rockett et al.^9^	
	Salib & Maximous^14^	
	Stack & Rockett^38^	
	Tewksbury et al.^39^	
	Weeke & Oberwittler^40^	
Children	*k* = 3, 9.1%	**NW were more likely to have children in *k* = 1:** Weeke and Oberwittler^40^
	Cerel et al.^8^	
	Demirel et al.^23^	
	Salib & Maximous.^14^	
Education level	*k* = 6, 18.2%	**NW were more likely to have higher education in *k* = 3:** Chavez‐Hernandez et al.^18^
	Cerel et al.^8^	
	Chavez‐Hernandez et al.^18^	Girdhar et al.^27^ O'Rourke et al.^36^
	Girdhar et al.^27^	
	Li et al.^32^	
	O'Rourke et al.^36^	
	Weeke & Oberwittler^40^	
Occupation	*k* = 11, 33.3%	**NW were more likely to be unemployed in *k* = 1:** Girdhar et al.^27^ **NW were less likely to be on household duties in** ***k*** **=** **1:** Ho et al.^29^
	Callanan & Davis^16^	
	Chaves‐Hernandez et al., 2006	
	Chia et al.^20^	
	Eisenwort et al.^24^	
	Foster^25^	
	Ho et al.^29^	
	Haines et al.^28^	
	Karbeyaz et al.^30^	
	Men et al.^34^	
	O'Connor et al.^35^	
	Tewksbury et al.^39^	
Socioeconomic status	*k* = 2, 6.1%	
	Carpenter et al.^17^	
	Girdhar et al.^27^	
Housing conditions	*k* = 13, 39.4%	**NW were more likely to live alone in *k* = 4:** Callanan & Davis^16^ Haines et al.^28^ Kuwabara et al.^10^ Men et al.^34^ **NW were more likely to have private housing in *k* = 1:** Men et al.^34^ **NW were more likely to live in urban areas in *k* = 1:** Chavez‐Hernandez et al.^18^
	Callanan & Davis^16^	
	Cerel et al.^8^	
	Chavez‐Hernandez et al.^18^	
	Cheung et al.^19^	
	Demirel et al.^23^	
	Foster^25^	
	Haines et al.^28^	
	Ho et al.^29^	
	Kuwabara et al.^10^	
	Men et al.^34^	
	Rockett et al.^9^	
	Salib & Maximous^14^	
	Stack & Rockett^38^	
Ethnicity	*k* = 9, 27.3%	**NW were more likely to be white in *k* = 3:** Cohen & Fiedler^22^ O'Rourke et al.^36^ Stack & Rockett^38^
	Callanan & Davis^16^	
	Cerel et al.^8^	
	Cheung et al.^19^	
	Chia et al.^20^	
	Cohen & Fiedler^22^	
	O'Rourke et al.^36^	
	Rockett et al.^9^	
	Stack & Rockett^38^	
	Tewksbury et al.^39^	
Place of origin (local vs immigrant)	*k* = 4, 12.1%	**NW were more likely to be locally born in *k* = 1:** Chia et al.^20^ **NW were less likely to be locally born in *k* = 1:** Carpenter et al.^17^
	Carpenter et al.^17^	
	Chia et al.^20^	
	Li et al.^32^	
	Wong et al.^41^	
Sexual orientation/gender identity	*k* = 1, 3.1%	**NW were more likely to be part of a sexual/gender minority in *k* = 1:** O'Rourke et al.^36^
	O'Rourke et al.^36^	
Religious beliefs	*k* = 3, 9.1% Chavez‐Hernandez^18^	**NW were more likely to have religious beliefs in *k* = 2:** Girdhar et al.^27^ Ho et al.^29^
	Girdhar et al.^27^	
	Ho et al.^29^	
Military career variables	*k* = 2, 6.1%	
	O'Rourke et al.^36^	
	Rockett et al.^9^	
**Clinical and psychosocial variables**		
Mental health problems	*k* = 17, 51.5%	**NW were less likely to have mental health problems in *k* = 4:** Chia et al.^20^ Demirel et al.^23^ Haines et al.^28^ Ho et al.^29^ **NW were more likely to have mental health problems in *k* = 2:** Kuwabara et al.^10^ Stack and Rockett^38^
	Carpenter et al.^17^	
	Cerel et al.^8^	
	Chia et al.^20^	
	Cheung et al.^19^	
	Demirel et al.^23^	
	Foster^25^	
	Freuchen & Grøholt^26^	
	Girdhar et al.^27^	
	Ho et al.^29^	
	Haines et al.^28^	
	Kuwabara et al.^10^	
	Lang et al.^31^	
	Li et al.^32^	
	Men et al.^34^	
	Rockett et al.^9^	
	Stack & Rockett^38^	
	Weeke & Oberwittler^40^	
Physical health problems	*k* = 14, 42.4%	**NW were more likely to have physical problems in *k* = 2:** Lang et al.^31^ Stack & Rockett^38^ **NW were less likely to have physical problems in *k* = 2:** Chia et al.^20^ Kuwabara et al.^10^
	Cerel et al.^8^	
	Chia et al.^20^	
	Cheung et al.^19^	
	Demirel et al.^23^	
	Foster^25^	
	Girdhar et al.^27^	
	Ho et al.^29^	
	Haines et al.^28^	
	Kuwabara et al.^10^	
	Lang et al.^31^	
	Li et al.^32^	
	Men et al.^34^	
	Rockett et al.^9^	
	Stack and Rockett^38^	
Contact with mental health services	*k* = 7, 21.2%	**NW were less likely to be known to mental health services in *k* = 2:** Haines et al.^28^ Salib & Maximous^14^
	Cerel et al.^8^	
	Cheung et al.^19^	
	Eisenwort et al.^24^	
	Foster^25^	
	Freuchen & Grøholt^26^	
	Haines et al.^28^	
	Salib & Maximous^14^	
Prior hospitalization for psychiatric or physical health reasons	*k* = 3, 9.1%	**NW were less likely to have prior hospitalizations in *k* = 1:** Haines et al.^28^
	Chia et al.^20^	
	Haines et al.^28^	
	Kuwabara et al.^10^	
Psychotropic medication	*k* = 1, 3.1%	
	Foster^25^	
Alcohol/substance abuse	*k* = 5, 15.2%	**NW were more likely to abuse alcohol/drugs in *k* = 1:** Stack & Rockett^38^ **NW were less likely to abuse alcohol/drugs in *k* = 3:** Chia et al.^20^ Lang et al.^31^ O'Rourke et al.^36^
	Cerel et al.^8^	
	Chia et al.^20^	
	Lang et al.^31^	
	O'Rourke et al.^36^	
	Stack & Rockett^38^	
Gambling problems	*k* = 1, 3.1%	**NW were more likely to have gambling problems in *k* = 1:** Chia et al.^20^
	Chia et al.^20^	
Distress	*k* = 1, 3.1%	**NW were more likely to have a higher level of distress in *k* = 1:** Haines et al.^28^
	Haines et al.^28^	
Hypochondria	*k* = 1, 3.1%	**NW were more likely to have a higher level of hypochondria in *k* = 1:** Haines et al.^28^
	Haines et al.^28^	
BMI	*k* = 1, 3.1%	
	Tewksbury et al.^39^	
Personality traits	*k* = 1, 3.1%	
	Freuchen & Grøholt^26^	
**Suicide‐related variables**		
Method	*k* = 24, 72.7%	**Methods that are more frequent in NW:** **Poisoning/overdose in *k* = 8:** Cohen & Fiedler^22^ Haines et al.^28^ Lang et al. ^31^ Li et al.^32^ Mejías‐Martín et al.^33^ O'Connor et al.^35^ Rockett et al.^9^ Stack and Rockett^38^ **Gas poisoning in *k* = 6:** Carpenter et al.^17^ Chia et al.^20^ Haines et al.^28^ Kuwabara et al.^10^ Li et al.^32^ Salib & Maximous^14^ **Hanging in *k* = 5:** Chia et al.^20^ Kuwabara et al.^10^ Li et al.^32^ O'Connor et al.^35^ Stack & Rockett^38^ **Firearms in *k* = 4:** Chynoweth^21^ Haines et al.^28^ Posener et al.^37^ Stack & Rockett^38^ **Drowning in *k* = 1:** Stack & Rockett^38^ **Asphyxiation in *k* = 1:** Cohen & Fiedler^22^ **Electrocution in *k* = 1:** Salib & Maximous^14^ **Plastic bags in *k* = 1:** Salib & Maximous^14^ **Sharp instrument in *k* = 1:** Kuwabara et al.^10^ **Non‐violent methods (unspecified) in *k* = 2:** Cheung et al.^19^ Men et al.^34^ **Methods that are less frequent in NW:** **Firearms in *k* = 2:** Rockett et al.^9^ Tewksbury et al.^39^ **Drowning in *k* = 2:** O'Connor et al.^35^ Salib & Maximous^14^ **Jumping from a height in *k* = 2:** Chia et al.^20^ Ho et al.^29^ **Vehicle or train in *k* = 1:** Carpenter et al.^17^ **Cutting wrists in *k* = 1:** Ho et al.^29^ **Poisoning in *k* = 1:** Haines et al.^28^ **Hanging in *k* = 1:** Haines et al.^28^
	Carpenter et al.^17^	
	Cerel et al.^8^	
	Chavez‐Hernandez et al.^18^	
	Cheung et al.^19^	
	Chia et al.^20^	
	Chynoweth^21^	
	Cohen & Fiedler^22^	
	Eisenwort et al.^24^	
	Foster^25^	
	Freuchen & Grøholt^26^	
	Girdhar et al.^27^	
	Ho et al.^29^	
	Haines et al.^28^	
	Kuwabara et al.^10^	
	Lang et al.^31^	
	Li et al.^32^	
	Men et al.^34^	
	Mejías‐Martín et al.^33^	
	O'Connor et al.^35^	
	Posener et al.^37^	
	Rockett et al.^9^	
	Salib and Maximous^14^	
	Stack & Rockett^38^	
	Tewksbury et al.^39^	
Precipitating circumstances	*k* = 13, 39.4%	**Precipitating factors that were more likely for NW:** **Relationship problems in *k* = 4:** Chia et al.^20^ Haines et al.^28^ Lang et al.^31^ Stack & Rockett^38^ **Financial or occupational problems in *k* = 4:** Chia et al.^20^ Demirel et al.^23^ Lang et al.^31^ Stack & Rockett^38^ **Death of a significant other in *k* = 2:** Haines et al.^28^ Stack & Rockett^38^ **Perpetrator of interpersonal violence in *k* = 1:** Weeke & Oberwittler^40^ **Precipitating factors that were less likely for NNW:** **Psychiatric disturbance in *k* = 2:** Haines et al.^28^ Kuwabara et al.^10^ **Physical health problems in *k* = 2:** Demirel et al.^23^ Kuwabara et al.^10^
	Cerel et al.^8^	
	Chia et al.^20^	
	Demirel et al.^23^	
	Eisenwort et al.^24^	
	Foster^25^	
	Freuchen & Grøholt^26^	
	Haines et al.^28^	
	Kuwabara et al.^10^	
	Lang et al.^31^	
	Li et al.^32^	
	Rockett et al.^9^	
	Stack and Rockett^38^	
	Weeke & Oberwittler^40^	
Past SA	*k* = 16, 48.5%	**NW were more likely to have previous SA in *k* = 3:** Beck et al.^15^ Freuchen & Grøholt^26^ O'Rourke et al.^36^ **NW were less likely to have previous SA in *k* = 2:** Chavez‐Hernandez et al.^18^ Ho et al.^29^
	Beck et al.^15^ Cerel et al.^8^	
	Chavez‐Hernandez et al.^18^	
	Cheung et al.^19^	
	Cohen & Fiedler^22^	
	Demirel et al.^23^	
	Foster^25^	
	Freuchen & Grøholt^26^	
	Girdhar et al.^27^	
	Ho et al.^29^	
	Haines et al.^28^	
	Kuwabara et al.^10^	
	Li et al.^32^	
	O'Rourke et al.^36^	
	Rockett et al.^9^	
	Weeke & Oberwittler^40^	
Past SI	*k* = 2, 6.1%	
	O'Rourke et al.^36^	
	Weeke & Oberwittler^40^	
Place of suicide	*k* = 7, 21.2%	
	Cerel et al.^8^	
	Chavez‐Hernandez et al.^18^	
	Kuwabara et al.^10^	
	Men et al.^34^	
	Rockett et al.^9^	
	Salib and Maximous^14^	
	Tewksbury et al.^39^	
Time of suicide	*k* = 5, 15.2%	
	Freuchen & Grøholt^26^	
	Ho et al.^29^	
	Kuwabara et al.^10^	
	Mejías‐Martín et al.^33^	
	Men et al.^34^	
Disclosed suicide intent	*k* = 4, 12.1%	**NW were more likely to have disclosed suicide intent in *k* = 2:** Weeke & Oberwittler^40^ **NW were less likely to have disclosed suicide intent in *k* = 1:** Callanan & Davis^16^
	Callanan & Davis^16^	
	Cerel et al.^8^	
	Haines et al.^28^	
	Weeke and Oberwittler^40^	
Evidence of planning	*k* = 1, 3.1% Weeke & Oberwittler^40^	**NW were more likely to have left evidence of planning in *k* = 1:** Weeke & Oberwittler^40^
Family history of suicide or SA	*k* = 1, 3.1%	
	Cerel et al.^8^	
Psychological state and behavior before the act	*k* = 2, 6.1%	**NW were less likely to be confused at the time of the act in *k* = 1:** Haines et al.^28^ **NW were more likely to show altered behavior before suicide in *k* = 1:** Freuchen & Grøholt^26^
	Haines et al.^28^	
	Freuchen & Grøholt^26^	
Alcohol or drug use at the time of suicide	*k* = 4, 12.1%	
	Cheung et al.^19^	
	Kuwabara et al.^10^	
	Rockett et al.^9^	
	Tewksbury et al.^39^	

Abbreviations: BMI, Body Mass Index; NNW, Non‐Note Writers; NW, Note Writers.

Regarding sex, 14 of the 28 studies (50%) investigating this variable reported significant differences between NW and NNW, although findings were inconsistent. Seven studies found that NW were more likely to be female^10^, ^19^, ^22^, ^31^, ^36^, ^38^, ^41^ whereas 5 studies reported that NW were more likely to be male.^17^, ^27^, ^32^, ^34^, ^35^ Two additional studies identified age‐by‐sex interaction effects, showing that women were more likely to leave suicide notes only among younger individuals,^29^ whereas men were more likely to do so among older individuals.^39^ Overall, the available evidence does not support a consistent association between sex and the likelihood of leaving a suicide note.

Regarding age, 11 of the 28 studies (39.3%) investigating this variable reported significant differences between NW and NNW, although the direction of the association was inconsistent. Five studies found that NW were more likely to be younger,^20^, ^28^, ^30^, ^34^, ^41^ 2 reported that they were more likely to be older,^36^, ^40^ and 1 found a higher likelihood among middle‐aged individuals.^38^ Three studies also identified significant age‐by‐sex interaction effects. Specifically, NW were more likely to be younger among women,^29^, ^32^ whereas they were more likely to be older among men.^32^, ^39^ Taken together, no consistent age profile emerged for individuals who left a suicide note.

Marital status significantly differentiated NW and NNW in 7 out of the 21 studies investigating this variable (33.3%). Overall, NW were more frequently unmarried, as reported in 5 studies.^20^, ^27^, ^28^, ^31^, ^38^ Two additional studies found that NW were less likely to be widowed.^22^, ^29^ Overall, the available evidence suggests that the absence of a current marital relationship, rather than a specific marital status category, may be associated with leaving a suicide note.

Housing conditions significantly differentiated NW and NNW in 6 studies (46.1%) out of the 13 investigating this variable. Four studies consistently reported that NW were more likely to live alone.^10^, ^16^, ^28^, ^34^ Additional findings indicated that NW were more likely to live in private housing^34^ and in urban areas.^18^ In summary, these findings suggest a possible association between suicide note writing and indicators of greater social isolation, although the available evidence remains limited.

Two studies (18.2%) out of 11 investigating occupation found significant differences: in one study, NW were more likely to be unemployed,^27^ whereas in the other, they were less likely to have household duties as their main occupation.^29^

Regarding ethnicity, 3 out of 9 studies (33.3%) investigating this variable reported that NW were more likely to be White.^22^, ^36^, ^38^

Three studies (50%) out of 6 investigating education level reported that NW tended to have a higher level of education.^18^, ^27^, ^36^

### Clinical and psychosocial variables

The most frequently investigated clinical and psychosocial variables were mental health problems (*k* = 17, 51.5%), physical health problems (*k* = 14, 42.4%), contact with mental health services (*k* = 7, 21.2%), and alcohol or substance abuse (*k* = 5, 15.2%). Variables examined in fewer than 5 studies are summarized in Table 2a.

Among the studies investigating mental health problems, six (35.3%) out of 17 reported significant differences between NW and NNW, although findings were inconsistent. Four studies found that NW were less likely to have psychological or psychiatric disorders,^20^, ^23^, ^28^, ^29^ whereas 2 studies reported the opposite association.^10^, ^38^ In summary, the available evidence does not support a consistent relationship between the presence of mental health problems and the likelihood of leaving a suicide note.

Four studies (12.1%) out of 14 investigating physical health problems reported significant differences between NW and NNW, but the findings were inconsistent. Two studies found that NW were more likely to have physical illnesses,^31^, ^38^ whereas 2 studies reported that they were less likely to have physical illnesses.^10^, ^20^ Therefore, no clear association emerged between physical health problems and suicide note writing.

Two studies (28.6%) out of 7 found that NW were less likely than NNW to have had previous contact with mental health services.^14^, ^28^ However, the limited number of investigations precludes firm conclusions.

Significant differences in alcohol or substance misuse were reported in 4 studies (80%) out of 5. Three studies found that NW were less likely to misuse alcohol or drugs,^20^, ^31^, ^36^ whereas one study reported the opposite association.^38^ The available evidence suggests that alcohol or substance misuse may be less common among NW, although this finding requires confirmation in future studies.

### Suicide‐related variables

The most frequently investigated suicide‐related variables were the method of suicide (*k* = 24, 72.7%), past SA (*k* = 16, 48.5%), precipitating circumstances (*k* = 13, 39.4%), place of suicide (*k* = 7, 21.2%), and time of suicide (*k* = 5, 15.2%). Variables examined in fewer than 5 studies are summarized in Table 2a.

The method of suicide was the variable most consistently different between NW and NNW. Among the 24 studies that investigated this characteristic, 16 (66.7%) reported that one or more suicide methods were significantly more frequent among NW. Specifically, NW were more likely to die by poisoning or overdose in 8 studies,^9^, ^22^, ^28^, ^31^, ^32^, ^33^, ^35^, ^38^ by gas inhalation in 6 studies,^10^, ^14^, ^17^, ^20^, ^28^, ^32^ by hanging in 5 studies,^10^, ^20^, ^32^, ^35^, ^38^ and by firearms in 4 studies.^21^, ^28^, ^37^, ^38^ Two additional studies reported a higher prevalence of non‐violent methods among NW,^19^, ^34^ whereas other methods (e.g., drowning, asphyxiation, electrocution, plastic bag suffocation, and sharp instruments) were each identified in only one study. Collectively, the available evidence suggests that suicide note writing is more frequently associated with suicide methods requiring greater preparation or allowing a longer interval between the suicidal decision and death.

Conversely, 9 studies (37.5% of the 24 studies investigating suicide method) reported that 1 or more methods were significantly less frequent among NW. Specifically, NW were less likely to use firearms in 2 studies,^9^, ^39^ drowning in 2 studies,^14^, ^35^ and jumping from a height in 2 studies.^20^, ^29^ Other methods, including vehicle or train‐related suicide,^17^ wrist cutting,^29^ poisoning,^28^ and hanging,^28^ were each reported by only one study. In summary, although findings for individual methods were not entirely consistent, the evidence suggests that suicide note writing is more commonly associated with methods involving greater planning and less frequently associated with highly immediate or impulsive methods.

Five of the 16 studies (31.3%) investigating previous SA reported significant differences between NW and NNW. Three studies found that NW were more likely to have a history of previous SA,^15^, ^26^, ^36^ whereas 2 studies reported the opposite association.^18^, ^29^ In summary, the available evidence does not support a consistent relationship between previous SA and the likelihood of leaving a suicide note.

Six studies (46.1%) out of 13 investigating precipitating circumstances reported that 1 or more precipitating circumstances were significantly more frequent among NW than NNW. The most frequently reported circumstances were relationship problems (*k* = 4^20^, ^28^, ^31^, ^38^) and financial or occupational problems (*k* = 4^20^, ^23^, ^31^, ^38^). Grieving the death of a significant other was reported in 2 studies,^28^, ^38^ whereas having perpetrated interpersonal violence was identified in 1 study.^40^ Overall, the available evidence suggests that suicide note writing may be more strongly associated with interpersonal and psychosocial stressors than with demographic or clinical characteristics.

Three of the 13 studies (23.1%) investigating precipitating circumstances reported that one or more precipitating factors were significantly less frequent among NW than NNW. Specifically, NW were less likely to have psychiatric disturbance identified as a precipitating circumstance in 2 studies,^10^, ^28^ and were less likely to have physical health problems reported as a precipitating circumstance in 2 studies.^10^, ^23^

Finally, no significant differences between NW and NNW were reported with respect to the place and time of suicide.

### Suicide attempts

Four studies out of 33 (12.1%) investigated individuals who had attempted suicide.^15^, ^40^, ^42^, ^43^ One of these studies^40^ included both SA and suicide deaths but did not report the findings separately according to the outcome. Therefore, results derived from this study should be interpreted as referring to a mixed sample. Variables investigated in only 1 study are reported in Table 2b.

**Table 2b:** Sociodemographic, clinical, psychosocial, and suicide‐related variables investigated in note writers (NW) and non‐note writers (NNW) in studies included in the systematic review with suicide attempt as outcome.

Variables	Studies that investigated the variable (*k*, %)	Significant differences between NW and NNW
**Sociodemographic variables**		
Gender	*k* = 2, 6.1% Huppert et al., 2021 Weeke & Oberwittler^40^	
Age	*k* = 3, 9.1%	**NW were more likely to be older in *k* = 1**:
	Huppert et al., 2021	Weeke & Oberwittler^40^
	Ruiz et al.^43^	
	Weeke & Oberwittler^40^	
Marital status	*k* = 3, 9.1% Huppert et al. 2021	
	Ruiz et al.^43^	
	Weeke & Oberwittler^40^	
Children	*k* = 2, 6.1% Huppert et al., 2021 Weeke & Oberwittler^40^	**NW were more likely to have children in *k* = 1:**
		Weeke & Oberwittler^40^
Education level	*k* = 1, 3.1% Weeke & Oberwittler^40^	
Housing conditions	*k* = 1, 3.1%	
	Ruiz et al.^43^	
Religious beliefs	*k* = 1, 3.1%	
	Ruiz et al.^43^	
**Clinical and psychosocial variables**		
Mental health problems	*k* = 3, 9.1%	
	Beck et al.^15^	
	Ruiz et al.^43^	
	Weeke & Oberwittler^40^	
Contact with mental health services	*k* = 1, 3.1%	
	Ruiz et al.^43^	
Alcohol/substance abuse	*k* = 1, 3.1%	**NW were less likely to abuse alcohol/drugs in *k* = 1:**
	Beck et al.^15^	
		Beck et al.^15^
Hopelessness	*k* = 2, 6.1%	**NW were more likely to have higher hopelessness in *k* = 1:**
	Beck et al.^15^	
	Ruiz et al.^43^	
		Ruiz et al.^43^
Physical pain	*k* = 1, 3.1%	
	Ruiz et al.^43^	
Vocabulary scores	*k* = 1, 3.1%	**NW had higher vocabulary scores in *k* = 1:**
	Beck et al.^15^	
		Beck et al.^15^
**Suicide‐related variables**		
Precipitating circumstances	*k* = 1, 3.1%	**Perpetrator of interpersonal violence in *k* = 1:**
	Weeke & Oberwittler^40^	
		Weeke & Oberwittler^40^
Past SA	*k* = 4, 12.1%	
	Beck et al.^15^	
	Huppert et al., 2021	
	Ruiz et al.^43^	
	Weeke & Oberwittler^40^	
Past SI	*k* = 3, 9.1%	
	O'Rourke et al.^36^	
	Ruiz et al.^43^	
	Weeke & Oberwittler^40^	
Disclosed suicide intent	*k* = 2, 6.1%	**NW were more likely to have disclosed suicide intent in *k* = 2:**
	Beck et al.^15^	
	Weeke & Oberwittler^40^	
		Beck et al.^15^ Weeke and Oberwittler^40^
Evidence of planning	*k* = 1, 3.1% Weeke & Oberwittler^40^	**NW were more likely to have left evidence of planning in *k* = 1:**
		Weeke & Oberwittler^40^
Family history of suicide or SA	*k* = 1, 3.1%	
	Ruiz et al.^43^	
Severity of SA	*k* = 2, 6.06%	**NW were more likely to have made a more serious suicide attempt in *k* = 2:**
	Huppert et al., 2021	
	Ruiz et al.^43^	
		Huppert et al., 2021 Ruiz et al.^43^

Abbreviations: NNW, Non‐Note Writers; NW, Note Writers.

Among sociodemographic variables, age and marital status were each investigated in 3 studies, whereas sex and parental status were investigated in 2 studies. Results were scarce and inconsistent. Only one study reported that NW were more likely to be older and to have children compared to NNW.^40^

Regarding clinical and psychosocial variables, mental health problems were investigated in 3 studies and hopelessness in 2 studies. One study found that NW reported higher levels of hopelessness,^43^ whereas another reported higher vocabulary scores and lower alcohol or substance misuse among NW.^15^ No consistent pattern emerged for the remaining clinical variables.

Among suicide‐related variables, previous SA were investigated in all 4 studies, whereas SI, disclosure of suicidal intent, and SA severity were examined in 2 or 3 studies. Two studies consistently found that NW had more severe SA.^42^, ^43^ Individual studies also reported higher suicidal intent,^15^ greater evidence of planning, more frequent disclosure of suicidal intent, and a higher likelihood of having perpetrated interpersonal violence among NW.^40^ In summary, the limited number of studies and their methodological heterogeneity preclude firm conclusions regarding the characteristics of suicide attempters who leave suicide notes.

### Quality of reporting

Of the 33 included studies, 15 (45.5%) were rated as good quality, 17 (51.5%) as fair quality, and 1 (3.0%) as poor quality (Table 3). Overall, the methodological quality of the included studies was moderate to high. Most studies clearly stated their research objectives, adequately described the study population, and employed well‐defined and consistently measured study variables. The most common methodological limitations concerned the absence of a sample size justification or power calculation, incomplete reporting of participation rates, limited adjustment for potential confounding variables, and the lack of information on assessor blinding. These shortcomings were particularly common among older studies and descriptive investigations of suicide notes, reflecting the methodological standards prevailing at the time they were conducted.

**Table 3:** The included studies using the quality assessment tool for observational cohort and cross‐sectional studies.

Study	1. Was the research question or objective in this paper clearly stated?	2. Was the study population clearly specified and defined?	3. Was the participation rate of eligible persons at least 50%?	4. Were all the subjects selected or recruited from the same or similar populations (including the same time period)? Were inclusion and exclusion criteria for being in the study prespecified and applied uniformly to all participants?	5. Was a sample size justification, power description, or variance and effect estimates provided?	6. For the analyses in this paper, were the exposure(s) of interest measured prior to the outcome(s) being measured?	7. Was the timeframe sufficient so that one could reasonably expect to see an association between exposure and outcome if it existed?	8. For exposures that can vary in amount or level, did the study examine different levels of the exposure as related to the outcome (e.g., categories of exposure, or exposure measured as continuous variable)?	9. Were the exposure measures (independent variables) clearly defined, valid, reliable, and implemented consistently across all study participants?	10. Was the exposure(s) assessed more than once over time?	11. Were the outcome measures (dependent variables) clearly defined, valid, reliable, and implemented consistently across all study participants?	12. Were the outcome assessors blinded to the exposure status of participants?	13. Was loss to follow‐up after baseline 20% or less?	14. Were key potential confounding variables measured and adjusted statistically for their impact on the relationship between exposure(s) and outcome(s)?	Rating
Beck et al.^15^	YES	YES	CD	NO	NO	NA	NA	NA	YES	NA	YES	CD	NA	NO	POOR
Callanan & Davis^16^	YES	YES	YES	YES	NO	NA	NA	YES	YES	NA	YES	CD	NA	YES	GOOD
Carpenter et al.^17^	YES	YES	YES	YES	NO	NA	NA	YES	YES	NA	YES	CD	NA	YES	GOOD
Cerel et al.^8^	YES	YES	YES	YES	NO	NA	NA	YES	YES	NA	YES	CD	NA	NO	FAIR
Chavez‐Hernandez et al.^18^	YES	YES	YES	YES	NO	NA	NA	YES	YES	NA	YES	CD	NA	NO	FAIR
Cheung et al.^19^	YES	YES	YES	YES	NO	NA	NA	YES	YES	NA	YES	CD	NA	YES	GOOD
Chia et al.^20^	YES	YES	YES	YES	NO	NA	NA	YES	YES	NA	YES	CD	NA	NO	FAIR
Chyno weth^21^	YES	YES	YES	YES	NO	NA	NA	YES	YES	NA	YES	CD	NA	NO	FAIR
Cohen & Fiedler^22^	YES	YES	YES	YES	NO	NA	NA	YES	YES	NA	YES	CD	NA	NO	FAIR
Demirel et al.^23^	YES	YES	YES	YES	NO	NA	NA	YES	YES	NA	YES	CD	NA	NO	FAIR
Eisenwort et al.^24^	YES	YES	CD	YES	NO	NA	NA	YES	YES	NA	YES	CD	NA	NO	FAIR
Foster^25^	YES	YES	CD	YES	NO	NA	NA	YES	YES	NA	YES	CD	NA	NO	FAIR
Freuchen & Grøholt^26^	YES	YES	NO	YES	NO	NA	NA	YES	YES	NA	YES	CD	NA	NO	FAIR
Girdhar et al.^27^	YES	YES	NO	YES	NO	NA	NA	YES	YES	NA	YES	CD	NA	NO	FAIR
Haines et al.^28^	YES	YES	YES	YES	NO	NA	NA	YES	YES	NA	YES	CD	NA	NO	FAIR
Ho et al.^29^	YES	YES	YES	YES	NO	NA	NA	YES	YES	NA	YES	YES	NA	NO	GOOD
Huppert et al., 2021	YES	YES	CD	YES	NO	NO	YES	YES	YES	NO	YES	CD	NA	YES	FAIR
Karbeyaz et al.^30^	YES	YES	YES	YES	NO	NA	NA	YES	CD	NA	YES	CD	NA	NO	FAIR
Kuwabara et al.^10^	YES	YES	YES	YES	NO	NA	NA	YES	YES	NA	YES	CD	NA	YES	GOOD
Lang et al.^31^	YES	YES	YES	YES	NO	NA	NA	YES	YES	NA	YES	CD	NA	YES	GOOD
Li et al.^32^	YES	YES	YES	YES	NO	NA	NA	YES	YES	NA	YES	CD	NA	YES	GOOD
Mejías‐ Martín et al.^33^	YES	YES	YES	YES	NO	NA	NA	YES	YES	NA	YES	CD	NA	NO	FAIR
Men et al.^34^	YES	YES	YES	YES	NO	NA	NA	YES	YES	NA	YES	CD	NA	YES	GOOD
O'Connor et al.^35^	YES	YES	CD	CD	NO	NA	NA	YES	YES	NA	YES	CD	NA	NO	FAIR
O'Rourke et al.^36^	YES	YES	YES	YES	NO	NA	NA	YES	YES	NA	YES	CD	NA	YES	GOOD
Posener et al.^37^	YES	YES	YES	YES	NO	NA	NA	YES	CD	NA	YES	CD	NA	NO	FAIR
Rockett et al.^9^	YES	YES	YES	YES	NO	NA	NA	YES	YES	NA	YES	CD	NA	YES	GOOD
Ruiz et al.^43^	YES	YES	YES	YES	NO	NA	NA	YES	YES	NA	YES	YES	NA	NO	GOOD
Salib & Maximous^14^	YES	YES	YES	YES	NO	NA	NA	YES	YES	NA	YES	CD	NA	NO	FAIR
Stack & Rockett^38^	YES	YES	YES	YES	NO	NA	NA	YES	YES	NA	YES	CD	NA	YES	GOOD
Tewksbury et al.^39^	YES	YES	YES	YES	NO	NA	NA	YES	YES	NA	YES	CD	NA	YES	GOOD
Weeke and Oberwittler^40^	YES	YES	YES	YES	NO	NA	NA	YES	YES	NA	YES	CD	NA	YES	GOOD
Wong et al.^41^	YES	YES	YES	YES	NO	NA	NA	YES	YES	NA	YES	CD	NA	YES	GOOD

Abbreviations: CD, Cannot Determine; NA, Not Applicable.

## DISCUSSION

This systematic review aimed to investigate the differences between NW and NNW across sociodemographic, clinical, psychosocial, and suicide‐related variables. Results suggest that leaving a suicide note is not associated with a distinctive sociodemographic or clinical profile. Rather, note‐writing may reflect a complex interaction between individual characteristics and the circumstances surrounding the suicidal act.

Across all the included studies, NW consistently represented a minority of individuals who died by suicide or engaged in a SA; thus, suicide notes seem to be the exception rather than the rule. This raises important questions about the representativeness of this subgroup and the extent to which findings based on note writers can be generalized to the broader population of individuals who die by suicide or attempt suicide. Although some variables showed recurring associations with note‐writing, the overall evidence was heterogeneous and often inconsistent across studies. Rather than solely reflecting a distinct psychological profile, leaving a suicide note may depend on the circumstances surrounding the suicidal act. For example, methods involving greater planning or a longer interval between the suicidal decision and death may provide both the opportunity and the cognitive resources necessary to compose a note. In this view, leaving a suicide note should be conceptualized as a behavior emerging from the interaction between personal characteristics, contextual factors, and the circumstances immediately preceding death. Importantly, our findings suggest that note‐leaving should not be viewed solely as a reflection of individual characteristics. Rather, it appears to emerge from the interplay between personal characteristics, situational opportunity, and the dynamics of the suicidal act.

As a speculative interpretation, we hypothesize that composing a suicide note may require a degree of cognitive organization, planning, and emotional regulation immediately before death. This interpretation is consistent with, though not directly tested by, linguistic analyses showing complex semantic organization^44^ and emotional expression.^45^ Thus we hypothesize that note‐leaving behavior may require a degree of cognitive and emotional organization that is inconsistent with disorganized or impulsive suicidal states. Acute psychosis, severe intoxication, or highly impulsive suicidal acts may reduce the opportunity or ability to produce a written communication. However, none of the included studies directly measured planning, cognitive organization, or emotional regulation, and this explanation should therefore be regarded as a hypothesis for future research rather than an established finding.

Additionally, the lower level of psychopathology and the lack of contact with mental health services found in NW might reflect low detection by healthcare services, or differences in help‐seeking behavior, rather than an actual lower level of symptomatology. In registry‐ and autopsy‐based studies, the absence of a recorded diagnosis does not necessarily indicate the absence of clinically significant symptoms, as psychiatric disorders may remain undetected or subthreshold.^46^ Likewise, lack of contact with mental health services may result from barriers to care or differences in help‐seeking behavior rather than lower clinical need.^47^

These findings have important clinical implications. A common misconception, also reported among healthcare professionals, is that those who talk about killing themselves rarely die by suicide.^48^ On the contrary, a large proportion of people who die by suicide have given at least some kind of verbal clue or non‐verbal warning of their intentions. Although the presence of a suicide note, or the expressed plan to write one, has been hypothesized to be associated with planning or suicidal intentionality, with some studies linking higher intentionality to higher lethality,^49^ this association was not directly examined in the present review, and the absence of a note should not be taken as a reassurance, given that the majority of suicide decedents do not leave a note. Clinicians should be equally attentive to suicidal risk regardless of whether a note is present and should not allow the absence of a note to lower their level of concern.

These findings also have important methodological implications. Suicide notes remain one of the few first‐person sources available for studying the suicidal process, but they should not automatically be considered representative of all suicide decedents. Instead, they may preferentially reflect the experiences of individuals whose suicidal acts involved sufficient planning, opportunity, or intention to communicate with others.

Finally, the direction of findings among SA was overall consistent with that among suicides. If note‐writing is similarly associated with both SA and suicide death, future studies may be able to investigate this behavior in survivors of SA, allowing prospective assessment of psychological correlates that cannot be examined among suicide decedents. Some differences did emerge; however, these must be interpreted with caution, considering the small number of studies focusing on SA, the heterogeneity of their samples, the fact that some studies did not separate results by suicide‐related outcome, and the poor quality of reporting of Beck et al.^15^

### Strengths and limitations

To the best of our knowledge, this is the first systematic review to systematically examine and synthesize evidence on differences between NW and NNW across sociodemographic, clinical, psychosocial, and suicide‐related variables. The fact that the screening process was done by independent assessors is a strength of the present work, as well as the variety of languages in the inclusion criteria.

However, some limitations should be considered. First, the included studies spanned almost five decades, during which the social meaning, accessibility, and modalities of suicide communication may have substantially changed. Furthermore, this review did not distinguish between single and multiple note writers: as NW were more likely to have a history of previous SA, it is likely that some of them already left a note in the past. Single‐ and multiple‐note leavers might have some different features that this review was not able to grasp. Another limitation concerns the definition of a suicide note itself. Variability in how suicide notes were defined across decades, including differences in whether digital communications (e.g., text messages, emails, social media posts) were counted as notes, likely contributed to heterogeneity in prevalence estimates and sample composition. The review focused exclusively on traditional suicide notes and did not consider digital farewell communications, which have become increasingly common in recent years. Furthermore, PsycINFO, Scopus, and Web of Science were not searched; however, although additional databases were not searched, ProQuest indexes a broad range of multidisciplinary and psychological literature, thus maximizing retrieval. Additionally, the search strategy relied primarily on title‐field terms, which, while increasing precision, may have missed studies in which relevant comparisons between NW and NNW were not reflected in the title. This risk was partly mitigated by the broad scope of the search strings (see Appendix 1) and by screening at the abstract and full‐text level, but it cannot be fully excluded. Finally, no backward or forward citation searching (e.g., of reference lists or citing articles) was performed, which represents an additional limitation and may have resulted in the omission of eligible studies not captured by the database search.

Other limitations that should be discussed are not problems of this review itself, but they mainly concern inherent weaknesses of the included studies. One limitation concerns the fact that included studies are heterogeneous in terms of sample size, participant profiles (such as age and the eventual presence of a clinical condition), research time and place settings, and investigated variables, further hindering the possibility of generalization of results. Another limitation concerns the design of the included studies, as most of them were cross‐sectional or retrospective studies, based on the gathering of information from forensic medical or police archives, and from interviews with family members. Thus, the use of standardized and validated measures was not always implemented, leading to risks like recall bias and eventual misreporting. Furthermore, the majority of included studies relied on bivariate or descriptive analyses without adjustment for confounding variables, which limits the interpretability of observed associations.

### Future directions

While suicide notes provide valuable insights into the thoughts, emotions, motivations, and interpersonal concerns that accompanied the suicidal process,^2^ it is also important to take into consideration not only who writes them but also the loved ones who receive them. It might seem intuitive to assume that a suicide note might have an effect on the mental health of the person who receives it; for example, comforting and supportive messages might reduce some sense of guilt.^50^ Unexpectedly, existing evidence shows no difference between people who received a note and people who did not in terms of mental health outcomes. Instead, the most salient effect appears among people who expected some kind of communication by their loved one but did not receive it: unmet expectations are associated with worse mental health.^50^ This finding underscores that the psychological impact of a suicide note is not determined solely by its content, but also by the pre‐existing relational dynamic and the expectations of those who survive, a topic that is only marginally covered by existing literature.

## CONCLUSION

This systematic review provides a comprehensive synthesis of the available evidence on differences between suicide NW and NNW across sociodemographic, clinical, psychosocial, and suicide‐related variables. The findings do not support a simple answer to the question of whether NW are representative of all suicide decedents. On one hand, the majority of investigated variables did not show consistent significant differences between the two groups, suggesting that NW and NNW are not radically distinct populations. On the other hand, where differences did emerge, they pointed in a meaningful direction: NW tended to use less violent methods, showed less severe psychopathology, were less likely to be known to mental health services, and appeared to be engaged in a relatively more deliberate suicidal process. Taken together, these patterns may suggest, but do not establish, that suicide note‐leaving is more likely to occur in the context of suicidal acts characterized by greater planning, cognitive organization, and communicative intent.

These findings have both methodological and clinical implications. From a clinical point of view, the presence of a suicide note, or an expressed intention to write one, may be worth considering as a possible indicator of deliberate suicidal planning that warrants careful assessment, though this should not be treated as an established or validated marker. Conversely, the absence of a note should never be interpreted as reassurance, given that the majority of individuals who die by suicide do not leave one.

Suicide notes should therefore be regarded as a valuable, but potentially selective, window into the suicidal process rather than as a representative account of all suicide deaths.

## AUTHOR CONTRIBUTIONS

**Giulia Gotti**: Methodology; writing—original draft. **Federica Turolla**: Methodology. **Alessandra Bordessa**: Methodology; writing—original draft. **Claudia Bruni**: Supervision; writing—review and editing. **Fabio Madeddu**: Conceptualization; supervision; writing—review and editing. **Lorenzo Montali**: Conceptualization; supervision; writing—review and editing. **Guendalina Gentile**: Conceptualization, supervision, writing—review and editing. **Riccardo Zoja**: Conceptualization; supervision, writing—review and editing. **Riccardo Primavera**: Methodology. **Stefano Tambuzzi**: Conceptualization; supervision; writing—review and editing. **Cristina Cattaneo**: Conceptualization; supervision; writing—review and editing. **Raffaella Calati**: Conceptualization; methodology; writing—original draft; supervision; writing—review and editing.

## CONFLICT OF INTEREST STATEMENT

The authors declare no conflicts of interest.

## ETHICS APPROVAL STATEMENT

N/A.

## PATIENT CONSENT STATEMENT

N/A.

## CLINICAL TRIAL REGISTRATION

N/A.

## Supporting information

Appendix 1 ‐ Search strings.

Supporting file 1.

## Data Availability

Data sharing not applicable—no new data generated, or the article describes entirely theoretical research.
