# Supplementary material for: Who leaves a suicide note? A systematic review of differences between note writers and non‐note writers
Source: PCN Rep. 2026 Sep 30;5(4):e70414. doi: 10.1002/pcn5.70414 (PMC13625211; doi:10.1002/pcn5.70414)
Supplement: Supplementary file 1 [file PCN5-5-e70414-s001.docx]

**Identification of studies via other methods**

**Identification of studies via databases and registers**

Records identified from:

Citation searching (n = 1)

etc.

Records removed *before screening*:

Duplicate records removed

(n = 431)

Records identified from databases (n = 870):

PubMed (n = 212)

ProQuest (n = 658)

**Identification**

Records excluded (reviews, meta-analyses, editorials, protocols, and dissertations):

(n = 105)

Records screened

(n = 439)

Reports not retrieved

(n = 0)

Reports sought for retrieval

(n = 1)

Reports sought for retrieval

(n = 334)

Reports not retrieved

(n = 13)

**Screening**

Reports excluded:

Not related (n = 56)

Foreign language (n = 6)

Wrong study design (n = 63)

No NNW (n = 119)

Simulated notes (n = 43)

Data on overlapping sample (n = 2)

Reports assessed for eligibility

(n = 1)

Reports excluded:

(n = 0)

Reports assessed for eligibility

(n = 321)

Studies included in review

(n = 33)

Reports of included studies

(n = 33)

**Included**

*Consider, if feasible to do so, reporting the number of records identified from each database or register searched (rather than the total number across all databases/registers).

**If automation tools were used, indicate how many records were excluded by a human and how many were excluded by automation tools.

Source: Page MJ, et al. BMJ 2021;372:n71. doi: 10.1136/bmj.n71.

This work is licensed under CC BY 4.0. To view a copy of this license, visit <https://creativecommons.org/licenses/by/4.0/>
