# Supplementary material for: Who leaves a suicide note? A systematic review of differences between note writers and non‐note writers
Source: PCN Rep. 2026 Sep 30;5(4):e70414. doi: 10.1002/pcn5.70414 (PMC13625211; doi:10.1002/pcn5.70414)
Supplement: Supplementary file 2 [file PCN5-5-e70414-s002.docx]

PUBMED

("suicid* note*"[title] OR "suicid* letter*"[title] OR "leave letter*"[title] OR "note leav*"[title] OR "note writ*"[title] OR "farewell letter*"[title]) AND suicid*

PROQUEST

title("suicid* note*" OR "suicid* letter*" OR "leave letter*" OR "note leav*" OR "note writ*" OR "farewell letter*") AND suicid*
